# Preventive strategy for the clinical treatment of hip fractures in the elderly during the COVID-19 outbreak: Wuhan’s experience

**DOI:** 10.18632/aging.103201

**Published:** 2020-05-07

**Authors:** Jing Liu, Bobin Mi, Liangcong Hu, Yuan Xiong, Hang Xue, Wu Zhou, Faqi Cao, Mengfei Liu, Lang Chen, Chenchen Yan, Hui Li, Guohui Liu

**Affiliations:** 1 Department of Orthopedics, Union Hospital, Tongji Medical College, Huazhong University of Science and Technology, Wuhan 430022, China

**Keywords:** hip fracture, elderly, preventive strategy, COVID-19

## Abstract

Hip fractures in the elderly account for more than half of osteoporotic fractures and represent a substantial economic and social burden. Novel coronavirus pneumonia (COVID-19), which began to spread in December 2019, has created challenges in the management of elderly hip fracture patients, not only by influencing the choice of operation and postoperative rehabilitation methods, but also by generating new risks for the medical staff. During this period, our infection and orthopedic treatment unit in the center of the epidemic area effectively treated 82 elderly patients with hip fracture, and no cross-infection occurred. Therefore, our experience in prevention and treatment is worth recommending to frontline anti-epidemic personnel.

## INTRODUCTION

In December 2019, novel coronavirus pneumonia cases were first reported in Wuhan, Hubei province, and the infection spread rapidly throughout Hubei province and eventually to the whole country [1, 2]. At present, most countries in the world have a large number of people infected with the virus, which has become a serious threat to human safety. Novel coronavirus pneumonia is caused by the 2019 novel coronavirus (SARS-CoV-2) [3]. On February 11, 2020, the World Health Organization announced an official name for the disease: COVID-19. SARS-CoV-2 has been classified as a lineage of β-coronavirus, and has characteristics typical of the coronavirus family. A recent study indicated that SARS-CoV-2 is very similar to another coronavirus carried by bats, leading to the speculation that bats may have hosted the novel virus [4].

The population is generally susceptible to SARS-CoV-2, with an incubation period of 1-14 days. COVID-19 patients are already infectious in the incubation period when they have no specific symptoms, creating great obstacles to the early detection of SARS-CoV-2 carriers and the early implementation of strict isolation measures [5]. The transmission routes include droplet transmission, contact transmission, and possibly fecal-oral transmission and aerosol transmission, so both orthopedic patients and medical staff may become infected with SARS-CoV-2.

Hip fractures in the elderly (aged > 65 years) account for more than half of osteoporotic fractures [6], causing a huge economic and social burden. The death rate and disability rate due to hip fractures in the elderly are very high, and the 30-day mortality rate is greater than 5% [7]. In principle, active surgical treatment should be performed unless the patient’s health condition is very poor, the patient cannot tolerate the operation, the risk of death during the operation is very high or postoperative nursing would be very difficult. In the perioperative period, multidisciplinary comprehensive treatment is needed to improve patients’ exercise abilities and quality of life.

The outbreak of COVID-19 has presented new challenges in the management of fractures and the protection of medical staff. During this period, our infection and orthopedic treatment unit in the center of the epidemic area effectively treated 82 elderly patients with hip fracture, and no-cross infection occurred. Therefore, we would like to share our treatment experience for the reference of frontline medical personnel at present.

### Why should we pay more attention to the treatment of hip fractures in elderly patients with COVID-19?

The prognosis of COVID-19 is relatively poor for elderly patients, especially those who are already at much higher risk for mortality than younger patients due to basic diseases such as heart disease, hypertension, diabetes, etc. [8]. Hip fractures in the elderly should generally be treated surgically at an early stage to prevent complications related to staying in bed, except in patients who cannot tolerate or are unwilling to undergo surgery [6]. Elderly patients with hip fractures often need to stay in bed for half a year or more, which makes it difficult for them to discharge lower respiratory tract secretions, and thus alters the treatment process for diseases such as COVID-19. In addition, hypostatic pneumonia due to staying in bed may exhibit similar symptoms to COVID-19, increasing the difficulty of clinical diagnosis and treatment. Thus, the surgical treatment of hip fractures in elderly COVID-19 patients should enhance both the fracture recovery and the COVID-19 treatment. On the other hand, weakness, fevers, immune responses and other systemic manifestations associated with COVID-19 may interfere with incision healing and postoperative rehabilitation training. Furthermore, a recent study indicated that surgical stress may activate or aggravate the progression and mortality of COVID-19 [9]. Therefore, orthopedic doctors should comprehensively analyze each patient’s situation in order to create the most favorable treatment plan for the individual.

### Typical clinical features of COVID-19 and hip fractures in the elderly

A fever, cough and fatigue are the main symptoms of COVID-19, and are sometimes accompanied by nasal obstruction, a runny nose, sore throat and diarrhea [10]. Severe patients usually experience dyspnea within one week, and gradually develop refractory hypoxemia, acute respiratory distress syndrome, septic shock, acid-base metabolic imbalance and other manifestations [11]. The diagnosis of suspected cases should be based on both their distinct epidemiological histories and their clinical symptoms, especially for those who have been exposed to infected persons or relevant environments within 14 days. Confirmed cases should have additional pathogenic or serologic evidence of SARS-CoV-2 obtained using quantitative real-time PCR, gene sequencing or antibody analysis [12].

With increasing age, age-related factors such as poor visual sensitivity, nervous system disease, altered drug sensitivity, myasthenia, abnormal gait and poor balance will increase, thus increasing the risk of fractures due to falling. Clinically, hip fracture often manifests as a history of hip injury, hip pain or a shortening/rotating deformity of the affected limb [13].

### Emergency treatment of hip fractures in elderly patients

During the COVID-19 epidemic, patients who come to the emergency department should first be questioned about their etiology and epidemiological history. Doctors should also measure each patient’s body temperature to determine whether further treatment is needed. Any patient with a fever or respiratory symptoms should immediately be transferred to the designated medical institution and isolated. Based on clinical experience, mild cases of COVID-19 can be discharged with simply symptomatic treatment; otherwise, computed tomography (CT) examination is needed to ascertain the diagnosis.

Patients who need to be hospitalized for elective surgery should undergo pulmonary CT and SARS-CoV-2 nucleic acid and antibody tests immediately. Patients requiring immediate orthopedic surgery should also have their overall condition assessed by an expert panel. Pulmonary CT and SARS-CoV-2 antibody analyses are also necessary for accompanying family members. Any detection of positive results of SARS-CoV-2 should be reported immediately, and patients should be transferred to the designated hospital for further treatment before surgery. If the results of SARS-CoV-2 analysis suggest a negative diagnosis for a patient who has typical characteristics on pulmonary CT, the patient should immediately be admitted to the infection department for isolation, and an expert panel should be consulted to exclude the differential diagnosis; then, the necessity of elective surgery should be assessed again.

At the same time, if hip fracture is suspected in a received patient, the patient should be questioned about his/her medical history, basic diseases and medications, and should undergo an orthopedic physical examination to determine the injury mechanism (e.g., a fall; then the cause of the fall, the stress on the hip during the fall, etc.). Patients should be asked to lie on their back, straighten their legs, avoid sitting and prevent hip stress [14]. Elderly hip fracture patients who are willing and able to meet the requirements of operation should have hospitalization treatment arranged as soon as possible, and should undergo elective surgery [15].

### Therapeutic principles for hip fractures in elderly patients during the COVID-19 epidemic

Conservative treatment is only applicable to patients who are unable (due to severe underlying diseases) or unwilling to undergo surgery [16]. Conservative treatment cannot effectively reduce and repair the broken ends of the fracture, which may lead to delayed union or even non-union of the fracture. Staying in bed long-term increases the risk of respiratory system infections, deep vein thrombosis of the lower extremities, bedsores and so on [17]. During the outbreak of COVID-19, confirmed patients may be forced to choose conservative treatment because their poor lung condition would prevent them from tolerating surgery. Uninfected patients may be less willing to undergo surgical treatment because they are afraid of cross-infection during their hospitalization.

For patients who are not infected with COVID-19, surgery should be performed as soon as possible, because the 30-day mortality after surgery decreases from 6.5% to 5.8% when the wait time for surgery is less than 24 hours [7]. The risk of cross-infection with SARS-CoV-2 in the perioperative period should be strictly controlled during the operation, and safety precautions such as isolation treatment and the use of an independent regional operating room should be taken to accelerate the process of entering and leaving the hospital and to reduce the length of stay.

For patients with suspected or confirmed COVID-19 infections, full preparation should be made before the operation, the operation should be performed with close monitoring of vital signs, limb function should be recovered as soon as possible, discharge should occur as soon as possible after the SARS-CoV-2 has been eliminated, and rehabilitation should be performed outside the hospital.

### Choice of operation methods

In terms of operation methods, the first choice is hollow screw fixation, which can maintain the stability of the fracture. Dynamic hip screw (DHS) fixation can be used for patients in better physical condition. For displaced femoral neck fractures (Garden type III, IV), hip arthroplasty is the first choice. Hemi or total hip replacement should be chosen according to the patient’s age, physical condition, activity before injury, acetabulum wear and mental health [18]. For stable intertrochanteric fractures, a DHS or intramedullary nail is the best choice. For unstable intertrochanteric fractures, an intramedullary nail is the first choice. In the treatment of subtrochanteric fractures, intramedullary nail fixation is the first choice, and long nail fixation can be used if necessary [19, 20]. Regardless of which operation method is adopted, to improve the prognosis of the elderly, it is very important to shorten the operation time and minimize soft tissue injury, blood loss and complications.

### Pre-operation discussion

At our treatment unit, senior doctors from the orthopedic department presided over the pre-operation discussions, and experts from the respiratory, infection, anesthesia and other relevant departments participated in the consultations. Pre-operation discussions must clarify key issues such as the initial diagnosis, COVID-19 infection status, pulmonary function classification and infectivity, proposed operation mode, personnel needed for the operation, required surgical instruments and consumables, blood product infusion demand, antibiotic demand, etc. For severe COVID-19 patients, the first task should be to save the patients’ lives, and surgical treatment should be carefully implemented. Suspected and confirmed patients must wear surgical masks. Hospital transport requires medical staff to travel together to ensure that the shortest distance is followed in the fastest amount of time without stopping on the way.

### Anesthesia management during operation

In principle, general anesthesia should be used to anesthetize COVID-19 patients or suspected patients. A disposable filter should be placed between the tracheal tube and the respiratory circuit to reduce the pollution of the respiratory circuit. At our treatment unit, before induction, two pieces of wet gauze were used to cover the nose and mouth, oxygen was given through a mask, and 100% pure oxygen was recommended for all patients. General anesthesia patients were advised to undergo rapid anesthesia induction and tracheal intubation after total muscle relaxation. The anesthesiologist completed the endotracheal intubation at a long distance with the help of an assistant. After the intubation, the disposable appliance was discarded into a designated garbage can, which was not to be taken out of the operating room.

Routine electrocardiogram results, blood oxygen saturation, end-tidal carbon dioxide partial pressure, invasive arterial blood pressure, body temperature, urine volume, arterial blood gas levels and coagulation function were monitored. After the operation, we recommend sending patients to the intensive care unit isolation ward, and then removing the endotracheal tube after their general condition has stabilized. We used a closed endotracheal suction system. The tracheal tube should be removed under analgesia to reduce choking.

### Intraoperative management

All medical staff should follow the principles of standard prevention and three-level prevention (Table 1). The participants in the anesthesia and operation procedures should be as few as possible, and should avoid entering other operating rooms. During the operation, surgeons should be under level III protection, and anesthesiologists may adopt level II protection, but their heads and faces should be equipped with a screen to prevent infection during tracheal intubation. Indoor personnel should not leave the room during the operation, and outdoor personnel should not enter the infected room.

**Table 1:** Classification of protection levels.

**Classification**	**Content**
Protection level I	Suitable for pre-examination triage, fever and infection clinics. Involves wearing a disposable working cap, disposable surgical mask (N95 protective mask when contacting patients with an epidemiological history), working clothes, medical protective clothing (disposable protective clothing when necessary for pre-examination and triage) and disposable latex gloves when necessary, and washing hands thoroughly.
Protection level II	Suitable for medical staff engaging in diagnosis and treatment activities in close contact with suspected or confirmed patients. Involves wearing a disposable working cap, protective goggles/mask, medical protective mask, protective clothing, disposable latex gloves and disposable shoe covers, and washing hands thoroughly.
Protection level III	Applicable to medical personnel who may be exposed to aerosol from suspected or confirmed patients due to sputum aspiration, respiratory sampling, tracheal intubation, tracheotomy, etc. When such personnel are working under the possibility of being sprayed or splashed with respiratory secretions or other substances, they should wear a disposable working cap, protective mask (or comprehensive respiratory protective device or positive pressure type head cover), medical protective mask, preventive clothing, disposable latex gloves and disposable shoe covers, and should wash their hands thoroughly.

After the operation, the medical personnel leaving the operating room must first replace their gloves, remove their protective clothing and foot covers and discard them in the designated garbage can. After removing their gloves, personnel should wash their hands thoroughly with a disinfectant, remove their mask, protective eyepiece or screen, and then wash their hands under running water for two minutes after leaving the operating room. After the operation, the goggles and masks should be sterilized with disinfectant paper towels and wiped with clean gauze for reuse. All the operation personnel should leave the operating room after bathing and changing their clothes.

In this process, strict requirements should be followed for putting on and taking off protective equipment. Protective equipment should be put on in the following order: hand disinfection → putting on working cap → putting on medical protective mask → putting on goggles or face shield / eye protection medical mask → putting on isolation clothing or protective clothing → putting on shoe covers → putting on gloves. Protective equipment should be taken off in the following order: taking off shoe covers → taking off gloves → hand disinfection → taking off isolation clothing or protective clothing → hand disinfection → taking off goggles or face shield → hand disinfection → taking off medical protective mask → hand disinfection → taking off working cap → hand disinfection and hand washing → replacing medical protective mask and disposable working cap.

Medical personnel can apply for exemption from isolation if there was no accidental exposure during the whole process. Otherwise, the medical staff involved in the operation should be observed for 14 days, and if there are any suspected symptoms during the observation period, they should be isolated and treated without delay.

### Postoperative patient management

The management of personnel in the ward should be strengthened, and personnel should have their temperature checked before entering the ward. Daily protection should be emphasized during the epidemic period, and coveralls and medical surgical masks should be worn. Once suspected patients are identified, they should be isolated in a single room immediately, and the COVID-19 diagnosis procedure should be started [21]. If contact with patients’ blood, body fluids, secretions, excreta, vomitus or pollutants may occur, personnel should wear latex gloves and wash their hands after removing the gloves. Personnel who may be splashed with patients’ blood or body fluids should wear goggles or a protective face shield and impermeable protective clothing.

If a patient develops a fever after the operation, it is important to determine whether the fever is due to COVID-19 infection, trauma or the operation itself by comprehensively examining various inflammatory indexes (white blood cell, neutrophil, lymphocyte, C-reactive protein and procalcitonin levels), the drainage tube and the wound exudate, in addition to markers of COVID-19. If postoperative dyspnea and reduced blood oxygen saturation occur, serious complications such as pulmonary embolism should be excluded [22]. At the same time, stress ulcers, gastrointestinal bleeding, venous thrombosis and other complications should be prevented and treated [23].

Suspected or confirmed COVID-19 patients should continue to be treated in isolation with their body temperature continuously monitored. If a patient’s body temperature returns to normal for more than three days, the lung CT images reveal obvious absorption of inflammation, and negative results have been obtained for respiratory pathogenic nucleic acids two consecutive times (with an interval of at least one day between samplings), the patient can be regarded as clinically cured of COVID-19. Patients can be discharged normally after being clinically cured; however, to avoid the risk of virus transmission as much as possible, it is still recommended that patients continue to practice centralized isolation under medical observation for two weeks [24, 25].

### Rehabilitation of hip fractures in elderly patients

The overall goal of postoperative rehabilitation for elderly hip fracture patients is to restore motor function to the lower limbs as soon as possible. In patients who are able to bear exercise activities, rehabilitation exercise can be started within six hours after the operation [26], and the help of a multidisciplinary rehabilitation team can be provided. Early rehabilitation exercise can reduce complications such as pressure sores or deep vein thrombosis while also accelerating postoperative recovery and shortening the hospital stay [27]. Rehabilitation plans including aerobic training of the upper limbs can increase patients’ adaptation and utilization of oxygen. Based on the results of postoperative rehabilitation, the patient should increase his/her weight-bearing exercise and seek to enhance balance. Rehabilitation exercise outside the hospital under the guidance of doctors can improve physical function and quality of life.

### Summary

Elderly patients with hip fractures often have a variety of underlying diseases, and are prone to related complications. The outbreak of COVID-19 has created great challenges for orthopedic doctors managing such patients. According to our experience, standardized and effective diagnosis and treatment is the key to ensuring that elderly hip fracture patients recover as soon as possible in the context of the COVID-19 epidemic. We recommend these orthopedic surgical practices for global medical workers fighting against the COVID-19 epidemic, especially among elderly hip fracture patients.
